# Determinants of Stroke Mortality through Survival Models: The Case of Mettu Karl Referral Hospital, Mettu, Ethiopia

**DOI:** 10.1155/2022/9985127

**Published:** 2022-02-11

**Authors:** Dereje Gebeyehu Ababu, Azmeraw Misganaw Getahun

**Affiliations:** Department of Statistics, Faculty of Natural & Computational Science, Mettu University, Ethiopia

## Abstract

**Introduction:**

Every year worldwide, between five to six million deaths are associated with stroke; on average, one stroke-related death occurs every four minutes. In Ethiopia, stroke is a frequent cause of mortality and morbidity from noncommunicable diseases. Therefore, this study was aimed at determining factors associated to stroke mortality through survival models in Mettu Karl Referral Hospital.

**Methods:**

This study was conducted from September 1, 2014, to April 1, 2017, and encompassed 202 stroke patients at Mettu Karl Referral Hospital. The Cox semiparametric regression was used for analyzing survival analysis of stroke patients using R software.

**Results:**

A total of 202 stroke patients were included in the study, and among those patients, 72.8% and 27.2% were censored and died, respectively. According to the result of Cox semiparametric regression model, sex of patients, hypertension, baseline complication, and stroke type had significant effect on survival of the stroke patient at 5% significance level.

**Conclusion:**

The results from Cox semiparametric regression model indicated that sex of patients, hypertension, baseline complication, and stroke type were major factors related to the survival time of stroke patients. The researcher recommends that the people should be aware on the burden of those risk factors and well informed about the disease.

## 1. Introduction

Globally, stroke is the second leading cause of death above the age of 60 years and about 16 million new cases of stroke and 62 million stroke survivors were estimated in 2005, with deaths from stroke accounting for 9.7% of all global deaths, and this is expected to increase to over 23 million new stroke cases and 7.8 million stroke deaths by 2030 in the absence of significant global public health response [1].

The study conducted in south western Saudi Arabia showed that prestroke smoking, prestroke hypertension, poststroke disturbed consciousness poor mobility, and developing pulmonary embolism as significant predictors of in-hospital stroke mortality [2].

In Africa, the impact of stroke is increasing due to a rising prevalence of hypertension associated with a very poor level of awareness and control. African countries are undergoing an epidemiological transition driven by sociodemographic and lifestyle changes [3]. The burden of noncommunicable diseases (NCD), including cardiovascular risk factors, is increasing. Consequently, the incidence of stroke, a cardinal complication of cardiovascular risk factors, appears to be rising in Africa [4].

In Ethiopia, hypertension was the most important risk factor identified in 1990. However, during that time, computed tomography (CT) scan was not available to differentiate stroke subtypes and the modern approach in treating patients with strokes requires early CT scan as it is imperative to diagnose whether the stroke is ischemic or hemorrhagic [5].

Although Ethiopia is progressing towards universal health coverage, the country faces the double burden of both communicable and noncommunicable diseases [6]. According to the report of WHO in 2017, stroke deaths in Ethiopia reached 6.23%. Moreover, the age-adjusted death rate of stroke in Ethiopia was 89.82 per 100,000 of the population. Earlier reports showed that the future trend of stroke in Sub-Sahara Africa will increase over the coming years owing to poor healthcare and poor neurologic interventions [7].

Therefore, chronic diseases become major global public health problems, mainly, in developing countries. However, there is limited evidence on the magnitude and factors of stroke in Ethiopia using survival models, particularly, in Mettu Karl Referral Hospital, which is the aim of this study. Findings of this study were important evidence to the studied hospital and Oromia Regional Health Bureau to know the burden of stroke and determinant factors and plan appropriate preventive methods against stroke.

## 2. Methodology

### 2.1. Study Area

This study was conducted in Mettu Karl Referral Hospital. This study was conducted from the 1^st^ of September 2014 to the 1^st^ of April 2017 in Mettu Karl Hospital.

### 2.2. Study Design and Population

Retrospective data were gathered from a total number of 202 stroke disease patients diagnosed with stroke case patients in the medical and surgical wards from the 1^st^ of September 2014 to the 1^st^ of April 2017 in MCRH.

### 2.3. Variable in the Study

The dependent (outcome) variable in this study was the survival time measured (in days) from the start date of stroke treatment until the date of the patient's death or censor. The following are covariate variables in the study: sex, age, hypertension, cardiac disease, diabetes mellitus, stroke type, baseline complication, and drug type.

### 2.4. Method of Data Analysis

Survival analysis is a collection of statistical procedures for data analysis for which the outcome variable of interest is time until an event occurs. An initial step in the analysis of a set of survival data is to present numerical or graphical summaries of the survival times in a particular group. In summarizing survival data, the two common functions of applied are the survivor function and the hazard function [6].

The Kaplan-Meier estimator is a nonparametric statistic used to estimate the survival function from lifetime data.

The Kaplan-Meier estimator of the survivorship function (or survival probability) *S* (*t*) = *P* (*T* ≥ *t*) is defined as

(1) $$\begin{array}{c} \hat{S}\left(t\right)=\underset{t_{i}\leq t}{\prod }\frac{n_{i}-d_{i}}{n_{i}}=\underset{t_{i}\leq t}{\prod }1-\frac{d_{i}}{n_{i}}, \end{array}$$

where *t*_1_, *t*_2_, ⋯, *t*_*n*_ a set of survival time of *n* independent observations and *t*_(1)_ ≤ *t*_(2)_ ≤ ⋯≤*t*_(*m*)_,  *m* ≤ *n* is the survival time of the *m* distinct ordered death times; *d*_*i*_ is the number of individuals who failed (died) at time *t*_*i*_; and *n*_*i*_ is the number of individuals who are at risk of dying at time *t*_*i*_.

One of the most popular types of regression models used in survival analysis is the Cox proportional hazard model [8]. The authors proposed a semiparametric model for the hazard function that allows the addition of covariates, while keeping the baseline hazards unspecified, and only positive values with this parameterization the Cox hazard function are given by

(2) $$\begin{array}{c} h\left(t,x\right)=h_{o}\left(t\right)\exp\left(\overset{`}{\beta }x\right), \end{array}$$

where*h*_0_(*t*) is the baseline hazard function and is called the baseline hazard function; *x* = (*x*_1_,*x*_2_, ⋯,*x*_*p*_)′ is the values of the vector of explanatory variables; and *β*′ = (*β*_1_, *β*_2_, ⋯, *β*_*p*_) is a vector of regression coefficients. The main assumption of the Cox proportional hazard model is proportional hazards that means that the hazard function of one individual is proportional to the hazard function of the other individual; i.e., the hazard ratio is constant over time.

## 3. Result and Discussion

### 3.1. Descriptive Summaries

A total of 295 stroke patients were treated in the hospital during the study period from the 1^st^ of September 2014 to the 1^st^ of April 2017. Of total population, this study included 202 stroke patients for whom data for variables of interest are complete. Of all 202 stroke patients, 147 (72.8%) were censored or did not experience the event and 55 (27.2%) died. Average time duration for all patients was 6.05 with a standard deviation of 4.698, and the median and mean survival time of age were 6 and 7.168 days, respectively. The mean and median age of stroke patient were 62.56 and 65 days, respectively. The mean and median survival time from stroke were found to be 15.596 and 19 days, respectively. The mean and median survival time from stroke were found to be 15.596 19 days, respectively. The mean survival time of male and female was 9.1 days and 5.1 days, respectively. The minimum and maximum survival times observed in the data were 1 day and 24 days, respectively. The median survival time of female and male was 3 days and 6 days, respectively. Patients with hypertension stayed for 4 days of which 72.13% were death. However, patients with no hypertension stayed for 9 days on average of which 27.87% were death. Patients with baseline complication stayed for 4 days on average of which 67.3% were death (Table 1).

### 3.2. The Kaplan-Meier Estimate

**Figure 1:**
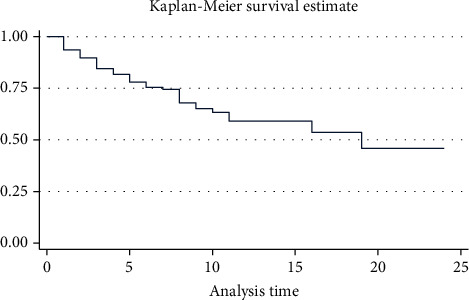
Overall Kaplan-Meier survivor function.

The estimate for overall Kaplan-Meier survivor function depicted that a relatively large number of the deaths occurred at the earlier days of antistroke treatment, and the same graph showed the decrement over a follow-up period (Figure 1).

**Figure 2:**
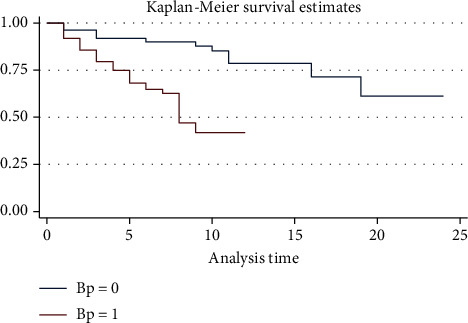
K-M plot survival of time-to-death by hypertension of stroke patients.

Kaplan-Meier estimates represented by the survival curves for without hypertension diseases are above those of the patients' complications with hypertension. This implied that the patients without hypertension have more chances of survival than those with hypertension (Figure 2).

### 3.3. Log-Rank Tests of Each Covariate

The log-rank test indicates that statistically there is a significant difference of survival experience among groups of gender, age, blood pressure (hypertension), and baseline complication. On the other hand, there are statistically no significant differences in survival/death experience among groups of the categorical covariates cardiac disease, diabetes mellitus, stroke type, and drug type. Accordingly, the mean survival time of male patient to death had been 9.1 days greater than that of female patients (5.1 days with 95% CI [8.04889, 10.3094]) (Table 2).

### 3.4. Univariable Analysis of Cox PH Regression Model

From the outputs in univariable analysis, we can observe that the covariate age of stroke patient (HR = 1.011731, *P* value = 0.0119), gender of patient (HR = 0.4123, *P* value = 1.58*e* − 06), hypertension (HR = 2.5510, *P* value = 2.40*e* − 06), stroke type (HR = 0.6152, 1.7325; *P* value = 0.014, 0.0961), and drug type and baseline complication (HR = 3.0710, *P* value = 4.84*e* − 08) are significant and hypertension, baseline complication, gender, and stroke type are highly significant in the univariable analysis. However, diabetes mellitus and cardiac are not significant factors for the death time at 5% level of significance (Table 3). This result is consistent with a previous study [3, 9] that age of patients was a significant factor in affecting the survival of stroke patients.

### 3.5. Multivariable Analysis of Cox PH Regression Model

Covariates which become insignificant in the multivariable analysis were removed from the model by using a stepwise elimination technique. Accordingly, cardiac disease and diabetes mellitus were excluded. In order to decide whether or not a variable is significant, the *P* value associated with each parameter has been estimated and variables that have *P* value less than 0.05 cut-point or 5% significance level are considered important variables and, hence, are included in the final model. Accordingly, sex, hypertension, baseline complication, and stroke type had significant effect on the survival of the stroke patient (Table 4). This result is supported by research conducted in China [5] in that hypertension was a significant factor in affecting the survival of stroke patients. The result is also consistent with that of a previous study [10] that sex of patients was a significant factor in affecting the survival of stroke patients. The finding of the study is also consistent with that of a previous study [9] that stroke mortality increased with age of patients.

### 3.6. Checking for the Linearity of Continuous Covariates in the Model

**Figure 3:**
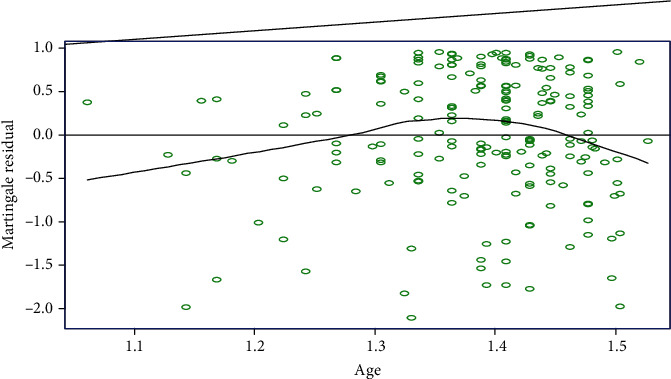
Plots of the martingale residuals against the covariate age.

For the age covariate, the plots show systematic patterns or trends and the resulting smoothed plots are not a straight line. Therefore, the plots of martingale residual confirm that age of a patient has no linear relationship with the survival time (Figure 3).

### 3.7. Checking of Proportional Hazard Assumption

The test of correlation (rho) is insignificant that indicates proportional hazards assumption is fulfilled. Variables including age, gender of patients, hypertension (blood pressure), baseline complication, drug type, and stroke type fulfilled the assumption because all the *P* values are greater than 0.05. In Schoenfeld residual, if the *P* value is greater than 0.05 it indicates that the Cox proportional hazard assumptions are fulfilled (Table 5).

#### 3.7.1. Diagnosis of the Model

In the likelihood ratio and significance of the final Cox PH model from the likelihood ratio test, it can be seen that the PH model is significant since *P* value is less than 5% (Table 6).

## 4. Conclusions and Recommendation

The objective of the study was to identify significant risk factors that affect survival of stroke patients who have been under follow-up at Mettu Karl Referral Hospital. For determining the risk factors for the survival time of stroke patients, a total of 202 patients were included in the study out of which 40.6% were females and 59.4% were males. Among those patients, 27.2% died and the rest were censored.

The Cox regression analysis showed that the major factors that affect the survival of stroke patients are hypertension, gender of the patients, and baseline complication which were strongly related to mortality, and based on the hospital outcome, the most common causes of death were hypertension and base line complication. However, there were variables that were significant at univariable stage of analysis but not at multivariate analysis stage. These were stroke type, drug type, and age of patients. Moreover, variables that were significant neither at univariable nor at multivariable analyses were gender, baseline complication, and hypertension. The result of this study also indicated that survival probability of a patient is not statistically different among groups classified by cardiac disease, diabetes mellitus, stroke type, and drug type.

The researchers recommend that the people should be aware on the burden of those risk factors and well informed about the disease. Finally, the researchers propose the concerned body special health management to improve the health of the society based on the result of the study.

## 5. Limitations

This research paper was limited to time from the date of the first diagnosis until the occurrence of the death or end of the study period for 202 stroke patients in case of Karl Hospital, Ilu Ababor Zone.

## Figures and Tables

**Table 1:** Baseline covariate characteristics with their time-to-death status.

Variables	Categories	Frequency	Death (%)	Censored (%)	Mean (days)	[95% CI]
Gender	Male	120 (59.4%)	34 (55.7%)	86 (61%)	9.179131	[8.04889, 10.3094]
	Female	82 (40.6%)	27 (44.3%)	55 (39%)	5.150107	[4.22813, 6.07208]
Blood pressure	Yes	123 (60.9%)	44 (72.13%)	77 (54.6%)	5.761887	[5.15259, 6.37119]
	No	79 (39.1%)	17 (27.87%)	64 (45.4%)	9.764186	[8.30564, 11.2227]
Cardiac disease	Yes	26 (12.9%)	11 (20%)	15 (10.2%)	8.069148	[5.14086, 10.9974]
	No	176 (87.1%)	44 (80%)	132 (89.8%)	7.770081	[6.85184, 8.68832]
Diabetes mellitus	Yes	15 (7.4%)	3 (5.45%)	12 (8.2%)	6.289487	[4.67067, 7.9083]
	No	187 (92%)	52 (94.54%)	135 (91.8%)	7.918552	[6.98651, 8.8506]
Stroke type	Hemorrhagic	70 (34.7%)	29 (52.73%)	41 (27.9%)	6.137696	[5.17646, 7.09893]
	Ischemic	117 (57.9%)	23 (41.82%)	94 (63.9%)	8.719683	[7.5657, 9.87366]
	Both	15 (7.4%)	3 (5.45%)	12 (8.2%)	4.584416	[3.25288, 5.91595]
Baseline complication	Yes	115 (56.9%)	37 (67.3%)	78 (53%)	5.391817	[4.83329, 5.95035]
	No	87 (43.1%)	18 (32.7%)	69 (47%)	9.92229	[8.54041, 11.3042]

**Table 2:** Log rank tests of each covariate.

Variable	Log rank test		
	Chi.sq	Df	*P* value
Gender	27.4	1	<0.001
Age	100	52	<0.001
Cardiac disease	0	1	0.988
Diabetes mellitus	0.9	1	0.341
Blood pressure (hypertension)	22.3	1	<0.001
Drug type	9.4	6	0.152
Baseline complication	31.4	1	<0.001
Stroke type	8.1	2	0.150

**Table 3:** Summary univariable (single covariate) Cox PH analysis.

Covariate	Category	Coef (*β*)	HR	se (*β*)	Wald	Pr (>∣*z*∣)	95% CI
Age		0.011662	1.011731	0.004634	6.33	0.012	[1.003, 1.0210]
Gender	0 female 1 male	Ref					
		-0.8860	0.4123	0.1845	23.05	<0.001	[0.2872, 0.5920]
Cardiac disease	0 no 1 yes	Ref					
		-0.003844	0.996164	0.273690	-0.014	0.989	[0.5826, 1.7030]
Diabetes mellitus	0 no 1 yes	Ref					
		0.2712	1.3116	0.3046	0.79	0.373	[0.7219, 2.3830]
Hypertension	0 no 1 yes	Ref					
		0.9365	2.5510	0.1986	22.24	<0.001	[1.7290, 3.7650]
Stroke type	0 (hemorrhagic) 1 (ischemic) 2 (both type)	Ref					
		-0.4857	0.6152	0.1976	-2.458	0.014	[0.4177, 0.9062]
		0.5496	1.7325	0.3303	13.44	0.096	[0.9068, 3.3101]
Baseline complication	0 no 1 yes	Ref					
		1.1220	3.0710	0.2056	29.78	<0.001	[2.052, 4.5950]

**Table 4:** Summary multivariable Cox PH analysis to the stroke data set from the MCRH.

Covariates	Category	Coef (*β*)	Haz. ratio	Std. Err. (*β*)	Wald	*P* > |*z*|	[95% Conf. Interval HR]
Age		0.0051853	1.0051988	0.0052631	0.985	0.325	[0.9949, 1.0156]
Gender	0 female 1 male	Ref					
		-0.7226692	0.4854547	0.1937196	-3.730	<0.001	[0.3321, 0.7096]
Blood pressure (hypertension)	0 no 1 yes	Ref					
		0.6713666	1.9569097	0.2145444	3.129	0.002	[1.2851, 2.9798]
Baseline complication	0 (no) 1 (yes)	Ref					
		0.5218596	1.6851584	0.2236382	2.333	0.020	[1.0871, 2.6122]
Stroke type	0 (hemo) 1 (ische) 2(both)	Ref		.			.
		-0.2105377 0.6232302	0.8101485 1.8649425	0.2094069 0.3409207	-1.005 1.828	0.315 0.068	[0.5374, 1.2213] [0.9560, 3.6380]

**Table 5:** Schoenfeld residual for each covariate.

Covariates	Categories	Rho	Chi^2^	Prob > chi^2^
Age		0.0462	0.2586	0.611
Gender	Female ref			
	Male	0.0304	0.1364	0.712
Hypertension	No ref			
	Yes	0.1115	2.3567	0.125
Baseline complication	No ref			
	Yes	0.0625	0.8123	0.367
Drug type	Nondrug (ref)			
	Drug type 1	-0.0974	1.3874	0.239
	Drug type 2	-0.0573	0.4887	0.485
	Drug type 3	-0.0111	0.0177	0.894
	Drug type 4	-0.0374	0.1989	0.656
	Drug type 5	-0.0445	0.2939	0.588
Stroke type	0 ref			
	Stroke type 1	-0.0229	0.0874	0.768
	Stroke type 2	0.0360	0.1898	0.663
Global test			8.4942	0.668

**Table 6:** The likelihood ratio and significance of the PH model.

Covariate	Coef (*β*)	HR	se (*β*)	Wald	*P* value
Factor (gender) 1	-0.712	0.491	0.193	-3.69	<0.001
Factor (BP) 1	0.654	1.923	0.208	3.15	0.002
Factor (baseline) 1	0.666	1.947	0.218	3.06	0.002

Likelihood ratio test = 49.8 on 3 DF; *P* = 8.87*e* − 11; *n* = 202; number of events = 147.

## Data Availability

The data used to support the findings of this study are available from the corresponding author upon request.

## Abbreviations

AIC: Akaike's Information Criterion
AIDS: Acquired immune deficiency syndrome
BIC: Bayesian Information Criterion
CT: Computed tomography
HIV: Human immunodeficiency virus
NCD: Noncommunicable diseases
WHO: World Health Organization.
